# Mass Spectrometry Supports That the Structure of Circulating Human Insulin-Like Factor 3 Is a Heterodimer

**DOI:** 10.3389/fendo.2020.00552

**Published:** 2020-08-28

**Authors:** Jakob Albrethsen, Anders Juul, Anna-Maria Andersson

**Affiliations:** ^1^Department of Growth and Reproduction, Rigshospitalet, University of Copenhagen, Copenhagen, Denmark; ^2^International Centre for Research and Research Training in Male Reproduction and Child Health (EDMaRC), Rigshospitalet, University of Copenhagen, Copenhagen, Denmark

**Keywords:** INSL3, LC-MS/MS, peptide hormone, structure, insulin family

## Abstract

The structure of the testicular peptide hormone insulin-like factor 3 (INSL3) has been the subject of discussion for more than a decade. Some studies support that the central C-domain of INSL3 is proteolytically removed and that INSL3 is secreted by the testicular Leydig cells into circulation as a small heterodimer consisting of an A- and a B-chain linked by two disulfide bridges. Other studies support that the INSL3 peptide remains uncleaved and that the predominant structure of circulating INSL3 is the larger pro-form. Furthermore, the structure of INSL3 could differ between species, and both structural forms of INSL3 could, in principle, be present in circulation. Recently, we have developed a mass spectrometry (MS)-based method for INSL3 in human serum that provides new information about the structure of circulating INSL3. Based on recent and newly presented data, we argue that in healthy men, the common, and probably the only, form of circulating INSL3 is the smaller AB heterodimer. For the first time, we demonstrate that the same analytical principle, with slight modifications, can also be applied to sera from other species, and we show that the INSL3 AB heterodimer is also present in serum from rodents. Improved understanding of the structure and biochemistry of circulating INSL3 could be valuable for the interpretation of INSL3 as a marker for reproductive and developmental disorders in humans and domesticated animals.

## Introduction

The peptide hormone insulin-like factor 3 (INSL3) is a member of the insulin/insulin-like growth factor/relaxin superfamily that in humans include insulin, insulin-like growth factor (IGF)-I and IGF-II, relaxin (RLN) 1-3, and INSL3-6 (1). The ten human peptides share the same motif of six cysteines that form three disulfide bridges, but otherwise do not show a significant sequence homology (2). In human males, INSL3 is secreted by the testicular Leydig cells, and in females by the ovarian Theca cells. INSL3 binds to the receptor Relaxin Family Peptide receptor 2 (RXFP2) that is expressed in several organs (1). INSL3 plays a key role in the fetal development and descent of the rodent testis (1), whereas mutations in genes encoding INSL3 and RXFP2 are rare causes of cryptorchidism in humans (3, 4). The postnatal function of INSL3 is poorly understood, but it may play a role in the regulation of male germ cell survival (5), bone metabolism (6), and muscle growth (7). Studies support that serum INSL3 is a promising clinical marker for Leydig cell functionality (1, 8, 9).

**Figure 1:**
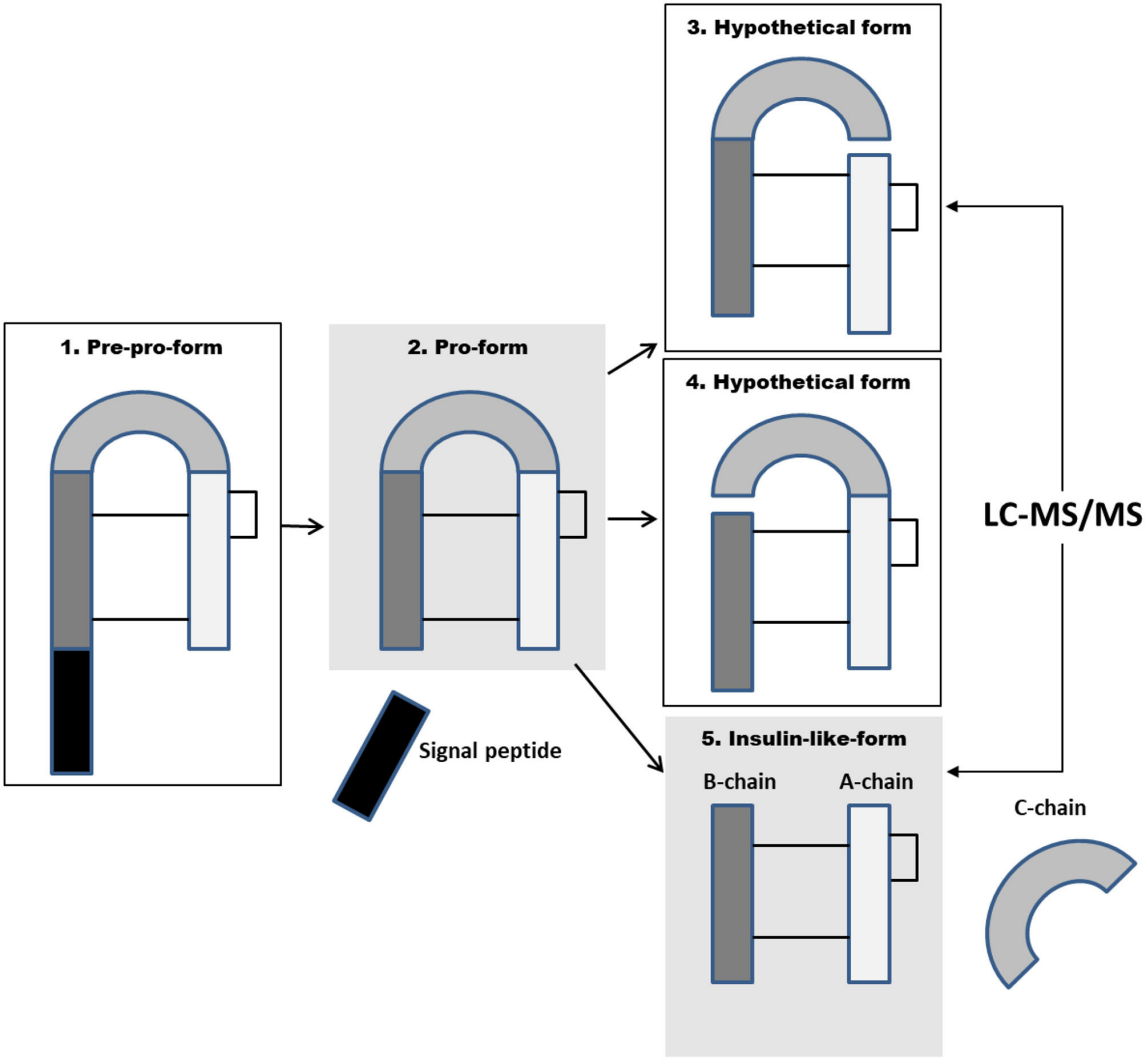
Maturation of the peptide hormone insulin-like factor 3 (INSL3). The black and gray boxes represent the four domains of the INSL3 peptide, including the signal peptide (black), B-chain (dark gray), C-domain (medium gray), and A-chain (light gray). The black lines represent the two disulfide bridges that connect the A- and B-chains, as well as the single internal disulfide bridge of the A-chain. During peptide maturation, the N-terminal signal peptide is first cleaved off from the “pre-pro-form.” This leaves the fully active “pro-form” consisting of the B-, C-, and A-chains. This form can undergo further proteolytic processing to produce the “insulin-like-form” where the central C-domain is cleaved out resulting in a heterodimer consisting of the A- and B-chains linked only by two disulfide bridges. Two forms have been identified in humans (gray boxes); isoform 5 has been identified in human plasma, and isoform 2 has been identified in human testicular tissues, whereas isoforms 3 and 4 represent hypothetical isoforms. Isoform 1 is presumed to be the original precursor peptide. The immunoassays would likely detect all shown possible human forms, whereas the MS-based assay would only detect human isoforms 3 and 5.

Members of the insulin peptide family are processed according to the classical mechanism for peptide hormones (10), whereby the N-terminal signal peptide is proteolytically cleaved from the nascent polypeptide chain during translation on ribosomes (Figure 1). In the case of the family member insulin, the resulting “pro-insulin” molecule undergoes an additional proteolytic cleavage step that removes the central C-peptide. The structure of circulating human insulin is therefore an ~6-kDa heterodimer that consists of two peptide chains (the A- and B-chains) that are covalently linked by two disulfide bridges only. In contrast, the central C-domain is not excised from the family members IGF-I and IGF-II, and both these peptides present in circulation as single chains of amino acids. Whether the central C-domain is cleaved from human INSL3, like the family member insulin, or whether the intact form of pro-INSL3 is released into circulation, like the family members IGF-I and IGF-II, is still being debated (11).

## Methods

**Figure 2:**
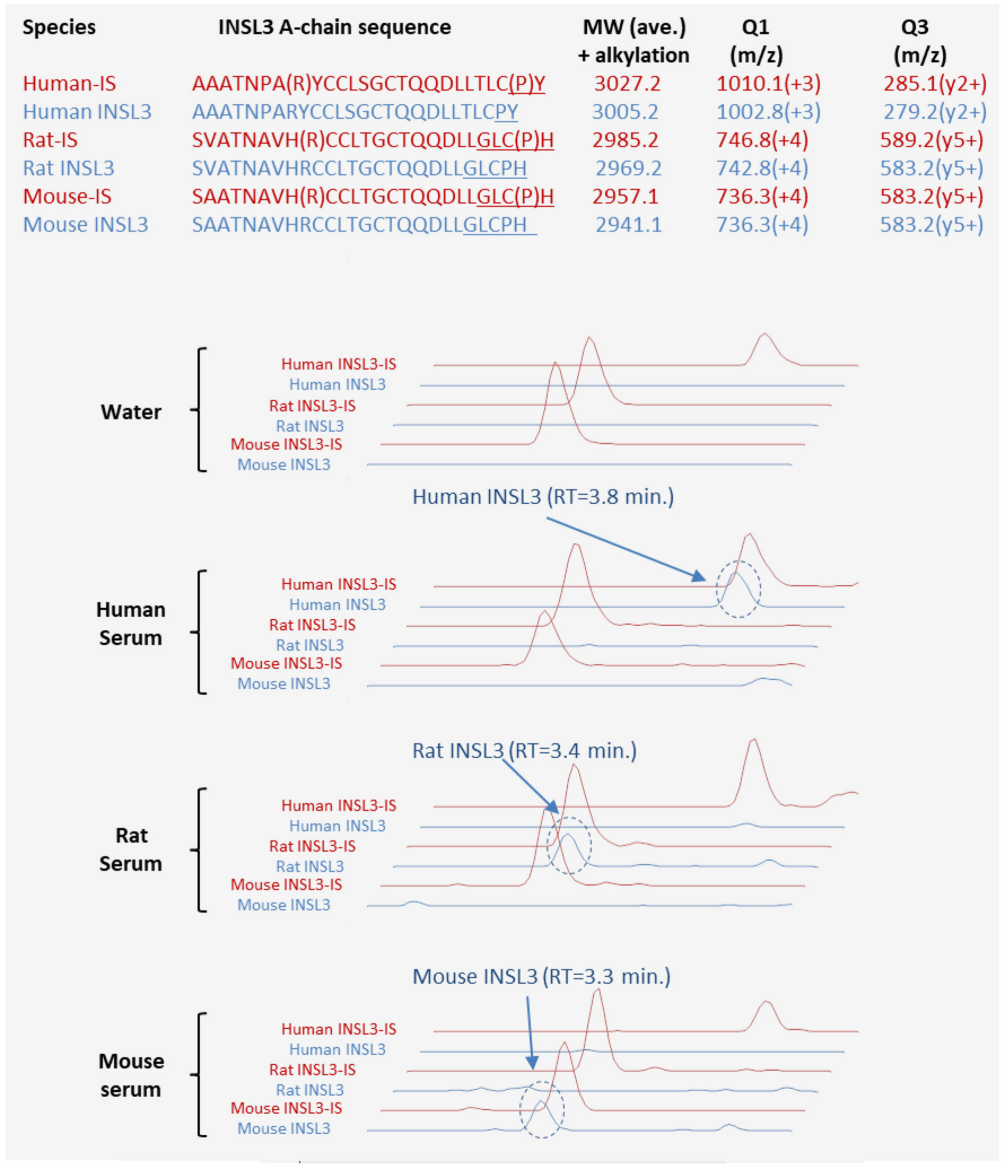
Parallel LC-MS/MS-based measurement of human, rat, and mouse INSL3. The top table shows the three amino acid sequences (blue) of the INSL3 A-chains from human, rat, and mouse and the sequences (red) of the corresponding stable-isotope-labeled (SIL) synthetic peptides used as internal standards (IS). The heavily labeled residues (R and P) of the SIL peptides are enclosed in brackets. The underscored sequence shows the fragment/quantifier ion for LC-MS/MS analysis. The molecular weight (MW) after alkylation and the mass-to-charge ratio (m/z) of the precursor (Q1) and fragment ion (Q3) are shown for each peptide. The chromatograms below show LC-MS/MS analysis of water, male human serum, male rat serum, and male mouse serum, respectively. The red chromatograms are the retention time (RT) three IS peptides (spiked into all samples), and the blue are the endogenous INSL3 peptides. Due to the analytical specificity with LC-MS/MS, the method can distinguish between INSL3 from the three species.

Serum was collected from three adult male Wistar rats and BL-6 mice. The serum was prepared, and INSL3 was measured as previously described (12), with slight modifications. For analysis of INSL3 in the rat and mouse serum, the mass spectrometry (MS) detection settings were modified to detect the species-specific isotope-labeled internal standards and the three endogenous INSL3 forms (see Figure 2 for details about peptides and MS transitions).

## The Structure of Human INSL3

The cDNA of human INSL3 was first identified in 1994, and it was apparent that human INSL3 includes a cleavable signal peptide at the N-terminus (13). In 2002, an “insulin-like” structure of INSL3 gained support from a study of extract from bull testes from where a 7-kDa INSL3 A-B heterodimer was purified and biochemically characterized (14). This pioneering study supported that in bulls, the C-domain is proteolytically removed from INSL3 and that the peptide hormone can be processed in a manner similar to insulin. However, the flanking cleavage sites of the INSL3 C-domain vary between species, and it has been proposed that in some mammals, possibly including humans, INSL3 is present in circulation as the larger pro-form or possibly as a mixture of the two forms (11, 15). Analyses of mammalian tissue have, in several cases, identified the larger pro-form (12–14 kDa) (16–21). This suggests that INSL3 is intracellularly stored, perhaps in granules, as the larger pro-form. INSL3 has also been identified in human tissue by shot-gun proteomics whereby proteins are first proteolytically digested by trypsin, and thereafter, the tryptic peptides are sequenced by liquid chromatography coupled to tandem mass spectrometry (LC-MS/MS). Several proteomics studies have identified peptide sequence corresponding to the INSL3 C-domain in human testis tissue (22). Importantly, the proteomics studies only show that amino acid sequence belonging to the INSL3 C-domain is present in tissue but do not clarify whether the C-domain is covalently connected to the A- and B-chains. In any case, the structure of INSL3 observed in studies of tissues does not reveal the structure in circulation.

In 2011, Minagawa et al. showed that the larger “pro-form” of INSL3 is secreted from Leydig cells into testicular venous blood in boars (23). This seems to conflict with an earlier study that identified the smaller INSL3 heterodimer in extracts from bovine testis (14). But, as argued by Minagawa et al. (23), the results from boars may not necessarily apply to other species where peptide maturation could be different. Pro-hormone convertase 1/3 (PC1/3) is responsible for proteolytic processing of many pro-hormones (24), including both insulin and RLN, and likely also INSL3. As pointed out by Minagawa et al., PC1/3 is not expressed in boar testes (25), and this likely explains why in boars the larger pro-form of INSL3 is released into circulation. In contrast, the testicular activity of PC1/3 in mice testis has been elegantly illustrated in a study where mice Leydig cells transfected with the human insulin transgene secreted the proteolytically excised insulin C-peptide (26). Thus, mice Leydig cells—as opposed to those in boars—contain the enzymatic machinery (including PC1/3) for the proteolytic removal of the C-domain from INSL3. Accordingly, it is plausible that mice may secrete the smaller and insulin-like form of INSL3, even if boars (primarily) secrete the larger pro-form of INSL3.

Until recently, circulating human INSL3 could only be measured by a well-validated and non-commercial research assay—a time-resolved fluorescent immunoassay (TRFIA) (27, 28), as well as by commercial immunoassays (available in both ELISA and RIA format). The LOD of the in-house TRFIA is 0.01 μg/L, and the intra- and inter-assay variations are <3 and <10%, respectively (29). The reported LOD of the commercial immunoassay according to the kit insert (Phoenix Pharmaceuticals) is 0.01 μg/L, and the intra- and inter-assay variations are <5 and 10%, respectively (30). However, the results are not directly comparable since the experimental designs to determine the assay performance differ between studies. In addition, there are instances of large interstudy differences (>10-fold) in the reported average concentration of circulating INSL3 in groups of healthy adult men using the same commercial kit. In our interpretation, this could indicate that this commercial kit may be less accurate. With the available immunoassays, the antibodies primarily bind to the INSL3 B-chain that is present in both forms of INSL3. Specifically, the antibodies do not bind to the isolated B-chain but only to the B-chain in the context of the A-B heterodimer and may possibly require epitopes on both the A- and B-chains. Thus, the immunoassays detect circulating INSL3 whether the C-domain is removed or not and thus cannot clarify which of the two forms are present, or predominant, in human blood. The B-chain is correctly folded in INSL3 with and without the C-domain, and both forms of INSL3 are active on the receptor RXFP2 (23, 31, 32).

We have recently developed an MS-based method for serum INSL3 (12). The LOD of the LC-MS/MS assay is 0.03 μg/L, the intra-assay variation is <9%, and the long-term variation (2 years) is <15% (9, 12). As opposed to the immunoassays, the MS-based method can only detect the smaller insulin-like form of INSL3 without the C-domain. This is because the MS method is designed to specifically detect the free A-chain after it is released from the B-chain by chemical reduction of the two disulfide bridges that form the only covalent link between the two chains that comprised the INSL3 A-B heterodimer (Figure 1). In the case of the larger pro-INSL3 molecule, chemical reduction does not release the A-chain since the A-chain is also covalently connected by a peptide binding to the C- and B-chains (Figure 1). Thus, with the MS method, the larger pro-INSL3 form does not contribute to the quantitation. If the pro-form of INSL3 is present in significant amounts in circulation, it must therefore be expected that the immunoassays would measure higher amounts of circulating INSL3 as compared with the MS method. This is not the case. We recently found a close agreement between the circulating level of INSL3 measured by TRFIA and LC-MS/MS in a direct comparison of human serum samples (12), and, secondly, the measured mean concentration of INSL3 is similar in two large studies using either the TRFIA or MS methods on serum cohorts from healthy adult men (9, 33). This supports that in healthy men, INSL3 is predominately present as the smaller heterodimer. Here, we utilize the same MS-based principle to detect INSL3 in the serum from male mice and rats, and the results show that the INSL3 A-B heterodimer is present also in the sera from male rodents (Figure 2). It should be emphasized that the presented results from rodents merely support the fact that the INSL3 AB heterodimer can be present in circulation in rodents. The results do not provide quantitative information nor do they exclude that other isoforms are commonly present. In addition, the measured level of INSL3 can vary substantially between strains and species, and further studies are needed to clarify the common structural forms of INSL3 in rodents.

## Discussion

The peptide hormone INSL3 is being explored as a diagnostic marker for developmental and reproductive disorders in both humans and domesticated animals. A detailed understanding of the peptide structure and biochemistry could be valuable for the reliable measurement and interpretation of circulating INSL3 levels. Recent information provided by MS now supports the fact that in men, the structure of circulating INSL3 is a heterodimer consisting of an A- and a B-chain linked by two disulfide bridges. Here, we also show that the INSL3 AB heterodimer is present in the sera from rats and mice. The structure of circulating INSL3 may be different in other species, like boars. To this extent, we would like to draw attention to six biochemical questions regarding circulating INSL3 that we believe also deserve attention: (1) Only two structural forms of INSL3 have been identified *in vivo*. In principle, there exist other possible forms including the two isoforms that would arise if the peptide was cleaved at only the B-C cleavage site or only at the C-A cleavage site (but not at both sites) (Figure 1). The two cleavage sites are different with the C-A cleavage site relying on multiple histidine residues that provide a basic charge, whereas the B-C cleavage site is a classical PC1/3 site. Other possibilities include the several isoforms that can be produced if the signal peptide is not cleaved off (not shown). The immunoassays would likely be capable of detecting all candidate human isoforms, whereas the MS-based method would only be capable of detecting the human isoforms from where the A-chain can be released by breaking of the disulfide bridges (Figure 1). If present in circulation, the longer pro-forms of INSL3 could potentially also have longer half-lives, and this may modulate the potency of the hormone. Evidence supports that INSL3 can be stored intracellularly, possibly in granules, as the larger pro-form, but little is known about the cleavage and the maturation process of INSL3. (2) By MS, we have detected low levels of INSL3 in the serum from a few women (unpublished results), and these preliminary results support the fact that in women, INSL3 is also present in the serum as the AB heterodimer. However, the structure of circulating INSL3 could, in principle, differ between women and men depending on the mechanism of peptide processing in Leydig vs. Theca cells. (3) It cannot be excluded that in men, the larger pro-form of INSL3 is occasionally secreted into the blood, for example, in connection with Leydig cell disorders. However, there is currently no evidence to support this, but it cannot be ruled out. (4) It is unknown if the excised C-domain of INSL3 is present in circulation and if it has a biological function (like the C-peptide from insulin). (5) Little is known about the possible degradation, half-life, and binding partners of circulating INSL3. Interestingly, *in vitro* studies support that insulin-degrading enzyme could play a role in INSL3 degradation, potentially in the testes (34). (6) The number and types of post-translational modifications (PTMs) are expanding rapidly thanks to novel MS-based analyses, and several hundred types of PTMs are now known (35). So far, no PTM has been identified on INSL3, but this does not exclude that the processing and biological activity of INSL3 is regulated by as yet unidentified PTMs.

In conclusion, MS analysis now supports that the structure of circulating INSL3 in men is an AB heterodimer. Additional structural and biochemical studies may further our understanding of the endocrine and clinical role of INSL3.

## Data Availability Statement

The raw data supporting the conclusions of this article will be made available by the authors, without undue reservation.

## Author Contributions

All authors listed have made a substantial, direct and intellectual contribution to the work, and approved it for publication.

## Conflict of Interest

The authors declare that the research was conducted in the absence of any commercial or financial relationships that could be construed as a potential conflict of interest.
